# Supplementary figures and images for: Prevalence of diabetes in pregnancy and microvascular complications in native Indonesian women: The Jogjakarta diabetic retinopathy initiatives in pregnancy (Jog-DRIP)
Source: PLoS One. 2022 Jun 15;17(6):e0267663. doi: 10.1371/journal.pone.0267663 (PMC9200361; doi:10.1371/journal.pone.0267663)

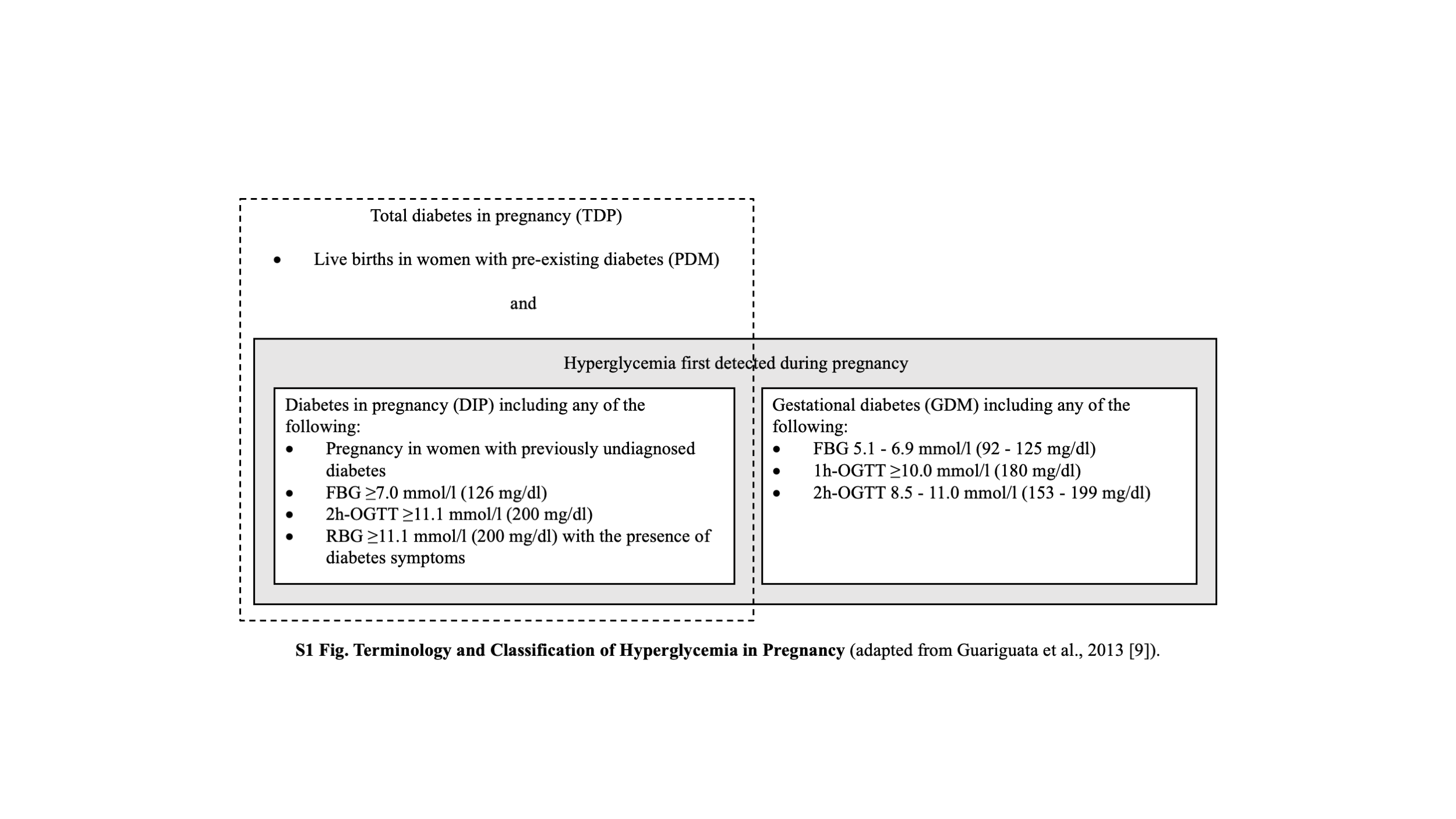

Supplement: S1 Fig — (TIFF) [file pone.0267663.s001.tiff]
