# Supplementary material for: Prevalence of diabetes in pregnancy and microvascular complications in native Indonesian women: The Jogjakarta diabetic retinopathy initiatives in pregnancy (Jog-DRIP)
Source: PLoS One. 2022 Jun 15;17(6):e0267663. doi: 10.1371/journal.pone.0267663 (PMC9200361; doi:10.1371/journal.pone.0267663)
Supplement: S1 Table — (PDF) [file pone.0267663.s002.pdf]

S1 Table. Comparison of demographic characteristics between normal, GDM and TDP groups

Characteristics	Normal	GDM	TDP	*p*-value*
**N**	478	146	7	
**Age group** [years], n (%)				0.546
<=25	116 (24.89)	31 (23.66)	1 (14.29)	
26 – 35	272 (58.2837	77 (58.78)	3 (42.86)	
>=35	78 (16.74)	23 (17.56)	3 (42.86)	
**Age** [years], median (IQR)	29 (26 – 34)	29 (26 – 35)	34 (26 – 39)	0.375
**Pregnancy stage at screening**, n (%)				***<0.001***
First trimester	***39 (8.35)***	***32 (22.54)***	***2 (28.57)***	
Second trimester	***272 (58.24)***	***66 (46.48)***	***3 (42.86)***	
Third trimester	***156 (33.40)***	***44 (30.99)***	***2 (28.57)***	
**Gestational age at screening** [weeks], median (IQR)	***26 (22 – 29)***	***23 (14 – 28)***	***23 (9-32)***	***0.001***
**Gravidity**, n (%)				0.480
Prime	154 (33.05)	53 (40.46)	3 (42.86)	
Second and third	280 (60.09)	68 (51.91)	4 (57.14)	
Fourth and above	32 (6.87)	10 (7.63)	0	
**Level of education,** n (%)				0.200
Never went to school	8 (1.72)	1 (0.77)	0	
Primary school	21 (4.51)	6 (4.62)	0	
Secondary school	356 (76.39)	87 (66.92)	5 (71.43)	
University degree	81 (17.38)	36 (27.69)	2 (28.57)	
**Household income/month** [IDR], n (%)				0.267
<1,000,000	54 (23.48)	30 (24.19)	2 (28.57)	
1,000,000 – 2,499,999	126 (54.78)	71 (57.26)	4 (57.14)	
2,500,000 – 4,999,999	45 (19.57)	15 (12.10)	1 (14.29)	
>=5,000,000	5 (2.17)	8 (6.45)	0	
**Residence**, n (%)				0.806
Urban area	363 (75.94)	114 (78.08)	6 (85.71)	
Rural area	115 (24.06)	32 (21.92)	1 (14.29)	
**BMI pre-pregnancy**, n (%)				0.073
Underweight	35 (8.29)	16 (13.33)	0	
Normal	236 (55.92)	74 (61.67)	2 (40.00)	
Overweight	108 (25.59)	20 (16.67)	1 (20.00)	
Obesity	43 (10.19)	10 (8.33)	2 (40.00)	
**BMI pre-pregnancy**, median (IQR)	23.64 (21.08 – 26.67)	22.56 (20.27 – 25.18)	26.27 (22.21 – 30.49)	0.107
**Systolic BP** [mmHg], median (IQR)	***110 (100 - 120)***	***116 (102 - 130)***	***115 (111 - 129)***	***0.015***
**Diastolic BP** [mmHg], median (IQR)	71 (68 - 80)	73 (69 - 80)	70 (64 - 80)	0.696

**Past smoker** [yes], n (%)	16 (3.49)	4 (3.10)	0	1.000
**Health insurance** [yes], n (%)	173 (73.31)	94 (75.20)	4 (57.14)	0.525
**History of medical conditions**:				
Hypertension [yes], n (%)	***17 (3.65)***	***11 (8.40)***	***2 (28.57)***	***0.006***
Dyslipidemia [yes], n (%)	52 (11.16)	8 (6.11)	0	0.214
Macrosomia baby in previous pregnancy [yes], n (%)	20 (7.38)	5 (7.69)	0	1.000
**Family history of diabetes** [yes], n (%)	52 (11.79)	***17 (14.53)***	***3 (60.00)***	***0.016***

BMI, body mass index; BP, blood pressure; IQR, interquartile range; TDP, total diabetes in pregnancy.

[*] *p*-value was estimated using Kruskal-Wallis test or Fisher's exact test as appropriate.
