# Supplementary material for: Prevalence of diabetes in pregnancy and microvascular complications in native Indonesian women: The Jogjakarta diabetic retinopathy initiatives in pregnancy (Jog-DRIP)
Source: PLoS One. 2022 Jun 15;17(6):e0267663. doi: 10.1371/journal.pone.0267663 (PMC9200361; doi:10.1371/journal.pone.0267663)
Supplement: S1 Appendix — (PDF) [file pone.0267663.s003.pdf]

CONFIDENTIAL<br>RESEARCH-ONLY PURPOSE

FORM : Diabetes in pregnancy

FACULTY OF MEDICINE, UNIVERSITAS GADJAH MADA
DEPARTMENT OF OPHTHALMOLOGY
DIABETIC RETINOPATHY IN PREGNANCY STUDY

BASIC MEDICAL DATA AND RISK FACTORS OF DIABETIC RETINOPATHY IN PREGNANCY FORM

**I. IDENTITY**				
1.	Study ID number	filled by researcher	☐☐☐☐	
2.	UR number		☐☐☐☐☐☐	
3.	FULL NAME	________________		
4.	DATE OF BIRTH	day/month/year	☐☐ ☐☐ ☐☐	
5.	CURRENT ADDRESS	________________<br>________________<br>________ POST CODE: ________		
6.	PHONE NUMBER	________ - ________	________________	
7.	MARITAL STATUS	1. Single<br>2. Married<br>3. Divorced<br>4. Widow	☐	
8.	ETHINICITY	1. Melayu<br>2. Chinese<br>3. Others	☐	
9.	RELIGION	1. Islam<br>2. Christian<br>3. Catholic<br>4. Hindu<br>5. Buddha	☐	
**II. SOCIOECONOMIC STATUS**				
1.	FAMILY INCOME PER MONTH?	1. < IDR 1 million<br>2. IDR 1 – 2,5 million<br>3. IDR 2,5 – 5 million<br>4. IDR 5 – 7,5 million<br>5. IDR 7,5 – 10 million<br>6. > IDR 10 million	☐	
2.	LEVEL OF EDUCATION	1. None<br>2. Primary school<br>3. Junior high graduate<br>4. Senior high graduate<br>5. University diploma or graduate<br>6. Postgraduate	☐	
3.	OCCUPATION	1. Housewife<br>2. Civil servant<br>3. Private sector<br>4. Entrepreneur<br>5. Farmer<br>6. Retired	☐	
4.	DO YOU HAVE HEALTH INSURANCE?	1. Yes<br>2. No	☐	

III. DIABETIC STATUS				
1.	TYPE OF DIABETES	1. Type 1<br>2. Type 2<br>3. Gestational	☐	
2.	TIME OF DIAGNOSIS	day/month/year	☐☐ ☐☐ ☐☐	
3.	AGE AT DIAGNOSIS		☐☐	YEARS
4.	INSULIN MEDICATION	1. Yes<br>2. No	☐	
	IF YES, FOR HOW LONG?		☐☐	MONTHS
5.	ORAL DIABETES MEDICINE	1. Yes<br>2. No	☐	
	IF YES, WHICH ONE(S) AND FOR HOW LONG?	Name of drugs (period of consumption)	1.<br>2.<br>3.	MONTHS
6.	OTHER MEDICATION?	1. Yes<br>2. No	☐	
	IF YES, please list the medications & indicate how long you have been taking them?	Name of drugs (period of consumption)	1.<br>2.<br>3.<br>4.<br>5.	MONTHS
7.	MANAGING DIET	1. Yes<br>2. No	☐	
8.	HAVE YOU HAD ANY KIND OF INFORMATION ABOUT DIABETES?	1. Yes<br>2. No	☐	
	HAVE YOU EVER EXPERIENCED:			
9.	DIABETIC COMA	1. Yes<br>2. No	☐	
10.	HYPOGLYCEMIA	1. Yes<br>2. No	☐	
11.	HEART DISEASE	1. Yes<br>2. No	☐	
12.	NEUROPATHY	1. Yes<br>2. No	☐	
13.	NEPHROPATHY	1. Yes<br>2. No	☐	
14.	FOOT ULCERS	1. Yes<br>2. No	☐	
15.	DIABETIC EYE DISEASES	1. Yes<br>2. No	☐	
	IF YES, WHEN?	day/month/year	☐☐ ☐☐ ☐☐	
	Please list any treatments you may have received for them		____________<br>____________	
16.	DO YOU THINK THAT DIABETES CAN DAMAGE YOUR EYES?	1. Yes<br>2. No	☐	
17.	HOW OFTEN DO YOU GO TO A DOCTORE REGARDING YOUR DIABETES?	1. Every week<br>2. Every month<br>3. Every 3 months<br>4. Every 6 months<br>5. Every year<br>6. Infrequent	☐	
18.	HOW OFTEN DO YOU HAVE YOUR EYES CHECKED?	1. Every month<br>2. Every 3 months<br>3. Every 6 months<br>4. Every year<br>5. Every 2 years<br>6. Infrequent	☐	
19.	WHEN WAS YOUR LAST EYE CHECK?	day/month/year	☐☐ ☐☐ ☐☐	

20.	HAVE YOU JOIN ANY DIABETES SUPPORT GROUPS?	1. Yes<br>2. No	☐	
21.	DO YOU HAVE FAMILY MEMBERS WITH DIABETES?	1. Yes<br>2. No	☐	
22.	IS ANYONE IN YOUR FAMILY SUFFERING VISION DISORDERS OR BLINDNESS BECAUSE OF DIABETES?	1. Yes<br>2. No	☐	
		**IV. PREGNANCY STATUS**		
1.	HAVE YOUR PREGNANCY BEEN CONFIRMED?	1. Yes<br>2. No	☐	
2.	FIRST DAY OF THE LAST MENSTRUAL PERIOD	day/month/year	☐☐ ☐☐ ☐☐	
3.	GESTATIONAL AGE		☐☐	WEEKS
4.	EXPECTED DUE DATE	day/month/year	☐☐ ☐☐ ☐☐	
5.	ATTENDANCE AT PRE-PREGNANCY CARE?	1. Yes<br>2. No	☐	
6.	NUMBER OF PREVIOUS PREGNANCIES		☐☐	
7.	NUMBER OF CURRENT CHILDREN		☐☐	
8.	DATE OF BIRTH OF YOUR CHILDREN	day/month/year	1.<br>2.<br>3.<br>4.<br>5.	
9.	BIRTH WEIGHT OF YOUR CHILDREN		1.<br>2.<br>3.<br>4.<br>5.	GRAMS
10.	GESTATIONAL AGE AT DELIVERY OF YOUR CHILDREN		1.<br>2.<br>3.<br>4.<br>5.	WEEKS OF PREGNANCY
11.	DID YOU HAVE ANY DISORDERS OR DIFFICULTIES DURING YOUR PREVIOUS PREGNANCY OR DELIVERY?	1. Yes<br>2. No	☐	
	If YES, please list		________<br>________	
12.	DID YOUR CHILDREN HAVE ANY HEALTH PROBLEMS?	1. Yes<br>2. No	☐	
	If YES, please list		________<br>________	
13.	DID YOU HAVE YOUR EYES CHECKED DURING YOUR PREVIOUS PREGNANCIES?	1. Yes<br>2. No	☐	
		**V. GENERAL HEALTH STATUS**		
1.	DO YOU SMOKE?	1. Yes<br>2. No	☐	
2.	IF YES, HOW MANY PACK OF CIGARETTE PER WEEK?		☐☐	PACKS
	HOW LONG HAVE YOU BEEN SMOKING?		☐☐	YEARS
3.	IF NO, DID YOU SMOKE?	1. Yes<br>2. No	☐	
	IF YES, WHEN DID YOU QUIT AND FOR HOW LONG DID YOU SMOKE?	Quit: month/year	☐☐ ☐☐	

		Lama merokok:	☐☐	YEARS
4.	DO YOU DRINK ALCOHOL?	1. Yes<br>2. No	☐	
	IF YES, HOW MANY GLASSES DO YOU HAVE PER WEEK?	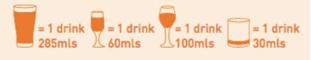	☐☐	ML/ WEEK
	HAVE YOU HAVE ANY OF THESE DISEASES?			
5.	HYPERTENSION	1. Yes<br>2. No	☐	
6.	HEART ATTACK	1. Yes<br>2. No	☐	
7.	KIDNEY DISEASE	1. Yes<br>2. No	☐	
8.	DYSLIPIDEMIA	1. Yes<br>2. No	☐	
9.	STROKE	1. Yes<br>2. No	☐	
10.	ASTHMA	1. Yes<br>2. No	☐	
11.	ANEMIA	1. Yes<br>2. No	☐	
12.	MIGREN	1. Yes<br>2. No	☐	
13.	ARTHRITIS	1. Yes<br>2. No	☐	
14.	OSTEOPOROSIS	1. Yes<br>2. No	☐	
15.	DID YOU HAVE ANY PREVIOUS SURGERY?	1. Yes<br>2. No	☐	
16.	IF YES, PLEASE LIST	Type of surgery:<br>1.<br>2.<br>3.<br>4.	Time:<br>1.<br>2.<br>3.<br>4.	
17.	YOUR BIRTH HISTORY	1. Normal<br>2. Premature<br>3. Low birth weight	☐	
18.	IF PRETERM, IN WHICH GESTATIONAL AGE WERE YOU BORN?		☐☐	WEEKS
19.	IF WITH LOW BIRTH WEIGHT, WHAT WAS YOUR BIRTH WEIGHT?		☐☐☐☐	GRAMS
**VI. PHYSICAL ACTIVITY**				
1.	HOW MANY DAYS A WEEK DO YOU WORK?		☐☐	DAYS
2.	HOW MANY HOURS A DAY DO YOU SLEEP?		☐☐	HOURS
3.	HOW MANY HOURS A DAY DO YOU SLEEP AT NIGHT?		☐☐	HOURS
4.	HOW FAR DO YOU WALK EACH DAY?		☐☐☐	METRES
5.	HOW MANY HOURS A WEEK DO YOU DO LIGHT EXERCISES?	(such as cleaning the house, walking)	☐☐	HOURS
6.	HOW MANY HOURS A WEEK DO YOU DO HEAVY EXERCISES?	(such as running,tennis, swimming, aerobic)	☐☐	HOURS
7.	HOW MANY HOURS DO YOU SIT EACH DAY?		☐☐	HOURS
8.	HOW MANY HOURS DO YOU RELAXING EACH DAY?		☐☐	HOURS
**VII. HISTORY OF EATING HABIT**				
1.	IN THE LAST YEAR, HOW MANY TIMES **A WEEK** DID YOU EAT:			
	RICE		☐☐	TIMES

	OTHER CARBS (WHEATS, CASAVAS, CORNS)		☐☐	TIMES
	FRESH VEGETABLES		☐☐	TIMES
	PRESERVED VEGETABLES		☐☐	TIMES
	MILK PRODUCTS (MILK, YOGHURT, CHEESE)		☐☐	TIMES
	FRESH FRUITS		☐☐	TIMES
	PRESERVED FRUITS		☐☐	TIMES
	FATTY MEAT		☐☐	TIMES
	PRESERVED MEATS (BEEF-CORNED, SAUSAGES, NUGGET)		☐☐	TIMES
	FISH		☐☐	TIMES
2.	HOW MANY TIMES A DAY DO YOU EAT RICE?		☐☐	TIMES
3.	WHAT DO YOU EAT FOR BREAKFAST?	1. Rice<br>2. Wheat/ cereal<br>3. Protein<br>4. Fruits<br>5. No breakfast	☐	
4.	WHAT DO YOU EAT FOR DINNER?	1. Rice<br>2. Wheat/ cereal<br>3. Protein<br>4. Fruits<br>5. No dinner	☐	
5.	HOW MANY HOURS DO YOU HAVE BETWEEN YOUR LAST MEALS AND SLEEP?		☐☐	HOURS
6.	HOW MANY TIMES A DAY DO YOU DRINK SWEET BEVERAGES?		☐☐	TIMES
7.	HOW MANY GLASSES A DAY DO YOU DRINK WATER?		☐☐	GLASSES
**VIII. DATA COLLECTION NOTES**				
1.	DATE OF DATA COLLECTION		☐☐ ☐☐ ☐☐	
2.	DATA COLLECTION METHOD	1. By patient<br>2. Interview	☐	
3.	INTERVIEWER	______________________________		
4.	SIGNATURE			
