# Supplementary material for: Prevalence of diabetes in pregnancy and microvascular complications in native Indonesian women: The Jogjakarta diabetic retinopathy initiatives in pregnancy (Jog-DRIP)
Source: PLoS One. 2022 Jun 15;17(6):e0267663. doi: 10.1371/journal.pone.0267663 (PMC9200361; doi:10.1371/journal.pone.0267663)
Supplement: S2 Appendix — (PDF) [file pone.0267663.s004.pdf]

RAHASIA<br>HANYA UNTUK PENELITIAN

FORM : Diabetes pada Kehamilan

FAKULTAS KEDOKTERAN UNIVERSITAS GADJAH MADA
BAGIAN ILMU KESEHATAN MATA
STUDI RETINOPATI DIABETIKA PADA KEHAMILAN

FORM KETERANGAN DASAR DAN FAKTOR RISIKO RETINOPATI DIABETIKA PADA KEHAMILAN

**I. IDENTITAS**				
1.	NO. IDENTITAS STUDI	Diisi oleh peneliti	☐☐☐☐	
2.	NO. REKAM MEDIS		☐☐☐☐☐☐	
3.	NAMA LENGKAP	______________________________		
4.	TANGGAL LAHIR	tanggal/bulan/tahun	☐☐ ☐☐ ☐☐	
5.	ALAMAT SAAT INI	______________________________<br>______________________________<br>________________KODE POS: ____________		
6.	NO. TELP/ HP	________ - ______________	______________________	
7.	STATUS PERKAWINAN	1. Tidak Menikah<br>2. Menikah<br>3. Cerai<br>4. Janda	☐	
8.	RAS	1. Melayu<br>2. Cina<br>3. Lain-lain	☐	
9.	AGAMA	1. Islam<br>2. Kristen<br>3. Katolik<br>4. Hindu<br>5. Budha	☐	
**II. STATUS SOSIAL EKONOMI**				
1.	BERAPA JUMLAH PENGHASILAN KELUARGA ANDA PER BULAN?	1. < 1 juta<br>2. 1 juta – 2,5 juta<br>3. 2,5 juta – 5 juta<br>4. 5 juta – 7,5 juta<br>5. 7,5 juta – 10 juta<br>6. > 10 juta	☐	
2.	TINGKAT PENDIDIKAN	1. Tidak sekolah<br>2. SD<br>3. SMP<br>4. SMA<br>5. D3/S1<br>6. Pasca Sarjana	☐	
3.	APAKAH PEKERJAAN ANDA?	1. Ibu Rumah Tangga<br>2. PNS<br>3. Swasta<br>4. Wiraswatsa<br>5. Petani<br>6. Pensiunan	☐	
4.	APAKAH ANDA MEMILIKI ASURANSI KESEHATAN?	1. Ya<br>2. Tidak	☐	

III. STATUS DIABETES				
1.	JENIS DIABETES APAKAH YANG ANDA MILIKI?	1. Tipe 1<br>2. Tipe 2<br>3. Gestasional	☐	
2.	KAPAN ANDA DIDIAGNOSIS MENDERITA DIABETES?	tanggal/bulan/tahun	☐☐ ☐☐ ☐☐	
3.	USIA BERAPA ANDA DIDIAGNOSIS MENDERITA DIABETES?		☐☐	TAHUN
4.	APAKAH ANDA MENGGUNAKAN INSULIN?	1. Ya<br>2. Tidak	☐	
	JIKA YA, SUDAH BERAPA LAMA?		☐☐	BULAN
5.	APAKAH ANDA MENGGUNAKAN OBAT DIABETES LAIN?	1. Ya<br>2. Tidak	☐	
	JIKA YA, APA NAMA OBAT TERSEBUT DAN SUDAH BERAPA LAMA?	Nama obat (lama penggunaan)	1.<br>2.<br>3.	BULAN
6.	APAKAH ANDA MENGGUNAKAN OBAT-OBATAN LAIN SAAT INI?	1. Ya<br>2. Tidak	☐	
	JIKA YA, SEBUTKAN DAN SUDAH BERAPA LAMA?	Nama obat (lama penggunaan)	1.<br>2.<br>3.<br>4.<br>5.	BULAN
7.	APAKAH ANDA MENGATUR DIET/ POLA MAKAN?	1. Ya<br>2. Tidak	☐	
8.	APAKAH ANDA PERNAH MENDAPAT PENGARAHAN MENGENAI DIABETES?	1. Ya<br>2. Tidak	☐	
	APAKAH ANDA PERNAH MENGALAMI:			
9.	KOMA DIABETIKUM	1. Ya<br>2. Tidak	☐	
10.	HIPOGLIKEMIA	1. Ya<br>2. Tidak	☐	
11.	PENYAKIT JANTUNG	1. Ya<br>2. Tidak	☐	
12.	KERUSAKAN SARAF AKIBAT DIABETES	1. Ya<br>2. Tidak	☐	
13.	GANGGUAN GINJAL AKIBAT DIABETES	1. Ya<br>2. Tidak	☐	
14.	ULKUS KAKI	1. Ya<br>2. Tidak	☐	
15.	PENYAKIT MATA TERKAIT DIABETES	1. Ya<br>2. Tidak	☐	
	JIKA YA, KAPAN?	tanggal/bulan/tahun	☐☐ ☐☐ ☐☐	
	TERAPI APA YANG ANDA DAPATKAN?		____________<br>____________	
16.	APAKAH MENURUT ANDA DIABETES DAPAT MERUSAK MATA/ PENGLIHATAN ANDA?	1. Ya<br>2. Tidak	☐	
17.	SEBERAPA SERING ANDA KONTROL KE DOKTER UNTUK DIABETES ANDA?	1. Setiap minggu<br>2. Setiap bulan<br>3. Setiap 3 bulan<br>4. Setiap 6 bulan<br>5. Setiap tahun<br>6. Tidak tentu	☐	
18.	SEBERAPA SERING ANDA MEMERIKSAKAN MATA ANDA?	1. Setiap bulan<br>2. Setiap 3 bulan<br>3. Setiap 6 bulan<br>4. Setiap tahun<br>5. Setiap 2 tahun<br>6. Tidak tentu	☐	
19.	KAPAN ANDA TERAKHIR MEMERIKSAKAN MATA ANDA?	tanggal/bulan/tahun	☐☐ ☐☐ ☐☐	

20.	APAKAH ANDA IKUT PERKUMPULAN DIABETES?	1. Ya<br>2. Tidak	☐	
21.	APAKAH DI KELUARGA ANDA ADA YANG MEMILIKI DIABETES?	1. Ya<br>2. Tidak	☐	
22.	APAKAH DI KELUARGA ANDA ADA YANG MENDERITA GANGGUAN PENGLIHATAN ATAU BUTA KARENA DIABETES?	1. Ya<br>2. Tidak	☐	
	**IV. STATUS KEHAMILAN**			
1.	APAKAH KEHAMILAN ANDA SUDAH DIKONFIRMASI?	1. Ya<br>2. Tidak	☐	
2.	HARI PERTAMA MENSTRUASI TERAKHIR (HPMT)	tanggal/bulan/tahun	☐☐ ☐☐ ☐☐	
3.	USIA KEHAMILAN		☐☐	MINGGU
4.	HARI PERKIRAAN LAHIR (HPL)	tanggal/bulan/tahun	☐☐ ☐☐ ☐☐	
5.	APAKAH ANDA MELAKUKAN PEMERIKSAAN PERSIAPAN KEHAMILAN?	1. Ya<br>2. Tidak	☐	
6.	JUMLAH KEHAMILAN SEBELUMNYA		☐☐	
7.	JUMLAH ANAK SAAT INI		☐☐	ORANG
8.	TANGGAL LAHIR ANAK SEBELUMNYA	tanggal/bulan/tahun	1.<br>2.<br>3.<br>4.<br>5.	
9.	BERAT LAHIR ANAK SEBELUMNYA		1.<br>2.<br>3.<br>4.<br>5.	GRAM
10.	USIA KEHAMILAN SAAT PERSALINAN ANAK SEBELUMNYA		1.<br>2.<br>3.<br>4.<br>5.	MINGGU
11.	APAKAH ADA GANGGUAN ATAU PENYULIT SAAT KEHAMILAN DAN PERSALINAN SEBELUMNYA?	1. Ya<br>2. Tidak	☐	
	JIKA YA, SEBUTKAN		____________<br>____________	
12.	APAKAH ADA MASALAH KESEHATAN PADA ANAK ANDA, BAIK SAAT INI ATAU DAHULU?	1. Ya<br>2. Tidak	☐	
	JIKA YA, SEBUTKAN		____________<br>____________	
13.	APAKAH ANDA MEMERIKSAKAN MATA ANDA PADA KEHAMILAN SEBELUMNYA?	1. Ya<br>2. Tidak	☐	
	**V. STATUS KESEHATAN UMUM**			
1.	APAKAH ANDA SAAT INI MEROKOK?	1. Ya<br>2. Tidak	☐	
2.	JIKA YA, BERAPA BUNGKUS ROKOK PER MINGGU?		☐☐	BUNGKUS
	BERAPA LAMA ANDA SUDAH MEROKOK?		☐☐	TAHUN
3.	JIKA TIDAK, APAKAH ANDA PERNAH MEROKOK?	1. Ya<br>2. Tidak	☐	
	JIKA YA, KAPAN ANDA BERHENTI DAN BERAPA LAMA ANDA MEROKOK?	Berhenti: bulan/tahun	☐☐ ☐☐	

		Lama merokok:		TAHUN
4.	APAKAH ANDA MINUM MINUMAN KERAS?	1. Ya<br>2. Tidak		
	JIKA YA, SEBERAPA BANYAK ANDA MINUM DALAM SEMINGGU?	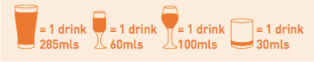 		ML/ MINGGU
	APAKAH ANDA MENDERITA/ PERNAH MENDERITA<br>PENYAKIT-PENYAKIT DI BAWAH INI?			
5.	HIPERTENSI	1. Ya<br>2. Tidak		
6.	SERANGAN JANTUNG	1. Ya<br>2. Tidak		
7.	PENYAKIT GINJAL	1. Ya<br>2. Tidak		
8.	KOLESTEROL TINGGI	1. Ya<br>2. Tidak		
9.	STROKE	1. Ya<br>2. Tidak		
10.	ASMA	1. Ya<br>2. Tidak		
11.	ANEMIA	1. Ya<br>2. Tidak		
12.	MIGREN	1. Ya<br>2. Tidak		
13.	ARTHRITIS/ SAKIT SENDI	1. Ya<br>2. Tidak		
14.	OSTEOPOROSIS/ SAKIT TULANG	1. Ya<br>2. Tidak		
15.	APAKAH ANDA PERNAH MENJALANI OPERASI?	1. Ya<br>2. Tidak		
16.	JIKA YA, SEBUTKAN DAN KAPAN	Jenis operasi:<br>1.<br>2.<br>3.<br>4.	Waktu:<br>1.<br>2.<br>3.<br>4.	
17.	STATUS SAAT ANDA DILAHIRKAN	1. Normal<br>2. Kurang bulan<br>3. Kurang berat		
18.	JIKA KURANG BULAN, BERAPA BULAN ANDA LAHIR?			BULAN
19.	JIKA KURANG BERAT, BERAPA BERAT ANDA WAKTU LAHIR?			GRAM

VI. AKTIFTAS FISIK

1.	BERAPA HARI DALAM SEMINGGU ANDA BEKERJA?			HARI
2.	BERAPA JAM DALAM SEHARI ANDA TIDUR? JAM			JAM
3.	BERAPA JAM ANDA TIDUR DI MALAM HARI?			JAM
4.	BERAPA JAUH ANDA BERJALAN SETIAP HARI?			METER
5.	BERAPA JAM ANDA BEROLAH RAGA RINGAN<br>DALAM SEMINGGU?	(misalnya membersihkan rumah, jalan-jalan pagi)		JAM
6.	BERAPA JAM ANDA BEROLAH RAGA BERAT DALAM<br>SEMINGGU?	(misalnya membersihkan jogging, tennis, berenang, aerobik)		JAM
7.	BERAPA JAM ANDA DUDUK SETIAP HARI?			JAM
8.	BERAPA JAM ANDA BERSANTAI SETIAP HARI?			JAM

VII. RIWAYAT POLA MAKAN

1.	DALAM SATU TAHUN TERAKHIR, SEBERAPA SERING **DALAM SEMINGGU** ANDA MAKAN :			
	NASI			KALI

	SUMBER KARBOHIDRAT LAIN (MISAL GANDUM, KETELA, JAGUNG)			KALI
	SAYURAN SEGAR			KALI
	SAYURAN AWETAN			KALI
	PRODUK SUSU (SUSU, YOGHURT, KEJU)			KALI
	BUAH-BUAHAN SEGAR			KALI
	BUAH-BUAHAN AWETAN			KALI
	DAGING BERLEMAK			KALI
	DAGING AWETAN (MISAL KORNET, SOSIS, NUGGET)			KALI
	IKAN			KALI
2.	BERAPA KALI DALAM SEHARI ANDA MAKAN NASI?			KALI
3.	APA YANG ANDA MAKAN KETIKA SARAPAN?	1. Nasi<br>2. Gandum/ cereal<br>3. Protein<br>4. Buah-buahan<br>5. Tidak sarapan		
4.	APA YANG ANDA MAKAN KETIKA MAKAN MALAM?	1. Nasi<br>2. Gandum/ cereal<br>3. Protein<br>4. Buah-buahan<br>5. Tidak makan malam		
5.	BERAPA JAM JARAK ANTARA MAKAN MALAM DAN TIDUR?			JAM
6.	BERAPA KALI DALAM SEHARI ANDA MINUM MINUMAN MANIS?			KALI
7.	BERAPA GELAS SEHARI ANDA MINUM AIR PUTIH?			GELAS
**VIII. KETERANGAN PENGAMBILAN DATA**				
1.	TANGGAL PENGISIAN KUESIONER			
2.	METODE PENGAMBILAN DATA	1. Diisi oleh pasien<br>2. Wawancara		
3.	PEWAWANCARA	____________________		
4.	PARAF			
